# Relationship between the sialylation level of anti-proteinase 3 antibodies and the activity of granulomatosis with polyangiitis (Wegener’s disease)

**DOI:** 10.1186/1479-5876-9-S2-P57

**Published:** 2011-11-23

**Authors:** Philippe Guilpain, Cécile Espy, Willy Morelle, Niloufar Kavian, Christian Pagnoux, Jean-Claude Michalski, Loïc Guillevin, Bernard Weill, Frédéric Batteux

**Affiliations:** 1 Médecine Interne A, Hôpital Saint-Eloi, Université Montpellier 1, Montpellier, France; 2 Médecine Interne, Hôpital Cochin, Université Paris 5, Paris, France; 3 Laboratoire d'immunologie, EA 1833, Université Paris 5, Cochin, Paris, France; 4 Unité de Glycobiologie Structurale et Fonctionnelle UMR8576, Université des Sciences et Technologies, Lille, France

## Background

Anti-proteinase 3 (PR3) antibodies (Abs) are associated with granulomatosis with polyangiitis (GPA), formerly called Wegener’s granulomatosis, a systemic necrotizing vasculitis. Their levels are not correlated with disease activity and cannot be used to predict clinical outcome. We assessed whether the glycosylation and sialylation levels of anti-proteinase 3 Abs could affect their pathogenicity and thus be correlated with the activity of GPA.

## Material and methods

Fourty-two sera from 42 patients with active or weakly active/inactive GPA were tested. Anti-PR3 Abs were assayed by ELISA and their levels of glycosilation and sialylation by enzyme-linked lectin assay. The glycosylation and sialylation levels of IgG purified from the sera of healthy donors and patients were determined by permethylation and mass spectrometry analysis of glycans. The neutrophil oxidative burst induced by purified IgG was assayed spectrofluorimetrically.

## Results

Patients with active or GPA exhibited lower sialylation ratio of anti-PR3 Abs than patients with weakly active or inactive disease. Sialylation ratio was inversely correlated with the activity indexes, BVAS and BVAS-WG (p<0.0001). The area under the ROC curve for the sialylation ratio of anti-PR3 Abs as a test to determine disease activity is 0.82 (p=0.0006). The characterization of N-glycans showed a decrease in 2,6-linked sialylated *N*-glycans and an increase in (dHex_1_Hex_3_HexNAc_4_(m/z 1836)) agalactosylated structures in purified IgG from patients with active GPA. The anti-PR3 Ab-induced oxidative burst of neutrophils was inversely correlated with the sialylation level of anti-PR3 IgG.

## Conclusion

The sialylation level of anti-PR3 Abs is associated with the clinical activity of GPA, through the modulation of the oxidative burst of neutrophils induced by these autoantibodies.

